# Pilot study for three-dimensional assessment of laminar pore structure in patients with glaucoma, as measured with swept source optical coherence tomography

**DOI:** 10.1371/journal.pone.0207600

**Published:** 2018-11-21

**Authors:** Kazuko Omodaka, Shigeto Maekawa, Guangzhou An, Satoru Tsuda, Yukihiro Shiga, Naoko Takada, Tsutomu Kikawa, Hidetoshi Takahashi, Hideo Yokota, Masahiro Akiba, Toru Nakazawa

**Affiliations:** 1 Department of Ophthalmology, Graduate School of Medicine, Tohoku University Graduate School of Medicine, Sendai, Japan; 2 Department of Ophthalmic Imaging and Information Analytics, Tohoku University Graduate School of Medicine, Sendai, Japan; 3 R&D Division, TOPCON Corporation, Tokyo, Japan; 4 Cloud-Based Eye Disease Diagnosis Joint Research Team, RIKEN, Wako, Japan; 5 Division of Ophthalmology, Tohoku Medical and Pharmaceutical University, Department of Medicine, Sendai, Japan; 6 Image Processing Research Team, RIKEN, Wako, Japan; 7 Department of Retinal Disease Control, Ophthalmology, Tohoku University Graduate School of Medicine, Sendai, Japan; 8 Department of Advanced Ophthalmic Medicine, Tohoku University Graduate School of Medicine, Sendai, Japan; Duke University, UNITED STATES

## Abstract

**Purpose:**

To develop a method to quantify, based on swept-source optical coherence tomography (OCT), the 3D structure of the laminar pores in patients with glaucoma.

**Methods:**

This retrospective study examined 160 laminar pores from 8 eyes of 8 cases: 4 normal subjects and 4 open-angle glaucoma (OAG) patients. We reconstructed 3D volume data for a 3 x 3 mm disc, using a method similar to OCT angiography, and segmented the structure of the lamina cribrosa. Then, we manually segmented each laminar pore in sequential C-scan images (>90 slices at 2.6-micron intervals) with VCAT5 (RIKEN, Japan). We compared the control and OAG subjects with the Mann-Whitney U test. Differences were considered significant at *p* < 0.05.

**Results:**

We found that the laminar pores of the OAG patients had a significantly smaller average cross-sectional area, smaller 3D volume (adjusted to the average thickness of the lamina cribrosa), and higher true sphericity, and lower principal value (P1, 2, 3) of the 3D structure data (all: *p* < 0.0001). The topographic distribution of damaged laminar pores was consistent with the damaged area of the macular map.

**Conclusion:**

We successfully developed a method to quantify the 3D structure of the laminar pores; providing a useful tool to assess lamina cribrosa-associated risk factors for glaucoma. These findings promise to benefit future investigations into the pathomechanisms of glaucoma.

## Introduction

Intraocular pressure (IOP) is the most important treatable risk factor for glaucoma, but many non-IOP risk factors have also been reported to contribute to glaucoma pathogenesis, including age [1, 2], myopia [2, 3], family history [4], abnormalities in the lamina cribrosa (LC) [5], low ocular perfusion pressure [6, 7], oxidative stress [8], inflammation [9], and lifestyle [5, 10]. Therefore, a significant body of evidence suggests that glaucoma should be considered a multifactorial disease. All forms of the disease share the same downstream pathology: glaucomatous optic neuropathy (GON), which is characterized by optic disc cupping, nerve fiber damage to the retinal ganglion cells (RGCs), and corresponding visual field loss.

The LC, which is located at the bottom of the optic disc cup, is composed of a series of sieve-like collagenous plates in the optic nerve head (ONH). It is generally believed that a crucial part of the underlying pathogenesis of glaucoma is axonal constriction, which is followed by disturbances in axoplasmic flow and axonal damage in the LC. This view is supported by evidence obtained with electron microscopy in human subjects [11]. Anti-BDNF immunohistochemistry has demonstrated that BDNF accumulates in the LC in a rodent model of acute intraocular pressure (IOP) increase [12]. Evidence has also been found that the LC can become thinned, with risk factors including high myopia [13] and pseudoexfoliation glaucoma [14]. Furthermore, technological progress in optical coherence tomography (OCT) has enabled the visualization of the structure of the LC, showing that defects in the LC [5] and thinning of the LC [15, 16] occur in glaucoma patients. We previously reported that thinning of the LC was already detectible in preperimetric glaucoma, and that decreased LC thickness was independently associated with the severity of glaucoma, cupping formation in the optic disc, and tissue blood flow in the ONH [17]. Taken together, these findings suggest that axonal damage in the LC plays a major role in glaucoma, and that LC thickness promises to be not only a biomarker of glaucoma, but also a source of new insights into the pathogenesis of glaucoma.

Previous findings on thinning of the LC in glaucoma suggested that laminar pore structure was also significantly altered, but detailed information on these alterations was lacking. Fundus photography has been used to examine “laminar dot signs” in glaucoma patients [18], and adaptive-optics SLO has been used to visualize laminar pores in living human subjects [19], although with limitations including a very small test area and a lack of data in the Z-axis. Another new technology, swept-source OCT (SS-OCT), which uses a high-penetration laser to visualize and observe the deep structure of the ONH in detail, has also been used to study the LC [20, 21]. New OCT technologies have demonstrated that in POAG eyes, the laminar pores become significantly elongated at the anterior surface of the LC, i.e., at a depth of 40 μm, but not at a depth of 80 μm [22]. This demonstrated that SS-OCT could be used to clearly show the structure of the laminar pores in living human subjects, and could improve our understanding of the details of glaucoma pathogenesis. This approach also avoids the possibility of artifacts being introduced during the process of fixation in postmortem specimens. However, 3D evaluation of the entire structure of the lamina pores with SS-OCT has not yet been reported.

In this study, we investigate the role of changes in the laminar pores during glaucoma pathogenesis by comparing normal subjects and patients with glaucoma. To assess the laminar pores, we reconstructed their entire 3D structure using a method that, first, obtained high-quality volume data based on quadruple SS-OCT images (similarly to OCT angiography; OCTA), and second, separated the top and bottom surfaces of the LC (as previously reported) [16]. Then, we segmented each pore in continuous C-scan images (with image processing software) and used this data to quantify and segment the pores in the 3D data. We found that 3D evaluation of the laminar pores was a useful method of understanding the condition of the axons running through the LC and provided potential new insights into the underlying pathomechanisms of glaucoma.

## Materials and methods

### Subjects

This cross-sectional study comprised a total of 8 eyes from 8 cases, including 4 normal subjects, and 4 open-angle glaucoma (OAG) patients. The inclusion criteria were good visual acuity (BCVA; best-corrected decimal visual acuity > 0.4) and a normal axial length (less than 28 mm). Patients with ocular diseases other than OAG, severe myopia (< -6 diopter), tilted myopic discs, or systemic diseases affecting the visual field were excluded from this study. There were no significant differences of the clinical characteristics, in age (Year, Normal: 69.0 ± 6.7, OAG: 68.0 ± 6.1, *p* = 0.90), BCVA (LogMAR, Normal: -0.08 ± 0.07, OAG: -0.10 ± 0.10, *p* = 0.85), IOP (mmHg, Normal: 12.0 ± 2.5, OAG: 13.0 ± 2.6, *p* = 0.59), refractive error (Diopter, Normal: -1.14 ± 2.9, OAG: -1.71 ± 2.9, *p* = 0.79), or axial length (mm, Normal: 24.5 ± 1.5, OAG: 24.6 ± 1.2, *p* = 0.90) between the groups.

Baseline clinical parameters were recorded for each patient. OAG was diagnosed based on findings of an open angle in a gonioscopic examination and glaucomatous optic neuropathy with corresponding abnormal visual field loss in an HFA (Carl Zeiss Meditec, Dublin, California, USA) examination, using the Swedish interactive threshold algorithm (SITA)-standard strategy of the 24–2 program. Only reliably measured data were used (i.e., with a fixation loss < 20%, false-positive errors < 15%, and false-negative errors < 33%). A glaucomatous visual field was defined, according to the Anderson-Patella criteria, [23] by one or more of the following: (1) a cluster of three points with probabilities of < 5% on the pattern deviation map in at least one hemifield including ≥ 1 point with probability of < 1% or a cluster of two points with a probability of < 1%, (2) glaucomatous hemifield test results outside the normal limits or (3) a pattern standard deviation beyond 95% of normal limits, as confirmed in at least 2 reliable examinations and the data of HFA in OAG was -8.1 ± 4.3 dB of mean deviation, 11.3 ± 3.3 dB in pattern standard deviation, and 76.8 ± 9.8% in visual field index.

Before pupil dilation, slit-lamp biomicroscopy and gonioscopy were performed to confirm a primary open angle in the patients. IOP was examined with Goldmann applanation tonometry on the same day as the OCT examination, without interrupting the use of OAG medication. Following pupil dilation with tropicamide (Midrin M, Santen Pharmaceutical, Osaka, Japan), stereoscopic examination of the ONH, fundus and disc photography, and ocular biometry (IOL Master; Carl Zeiss Meditec) were performed. Circumpapillary retinal nerve fiber layer thickness (cpRNFLT) was calculated with the included OCT software (3D OCT-2000, version 8.00), in 16 overlapping circular OCT images centered on the optic disc. We analyzed the OCT images from one eye of each patient. We excluded OCT images with quality less than 70. Abnormal RNFL thickness was defined as a > 99% difference from an intergenerational normalized database (cpRNFLT, Normal 105 ± 30.1 μm, OAG: 68.5 ± 18.7 μm). All the examination data were obtained within a 2-month period.

This study adhered to the tenets of the Declaration of Helsinki, and the protocols were approved by the Clinical Research Ethics Committee of the Tohoku University Graduate School of Medicine (study 2014-1-805). Participants provided their written informed consent to participate in this study. The ethics committee approved this consent procedure.

### Segmentation of laminar pores

**Fig 1:**
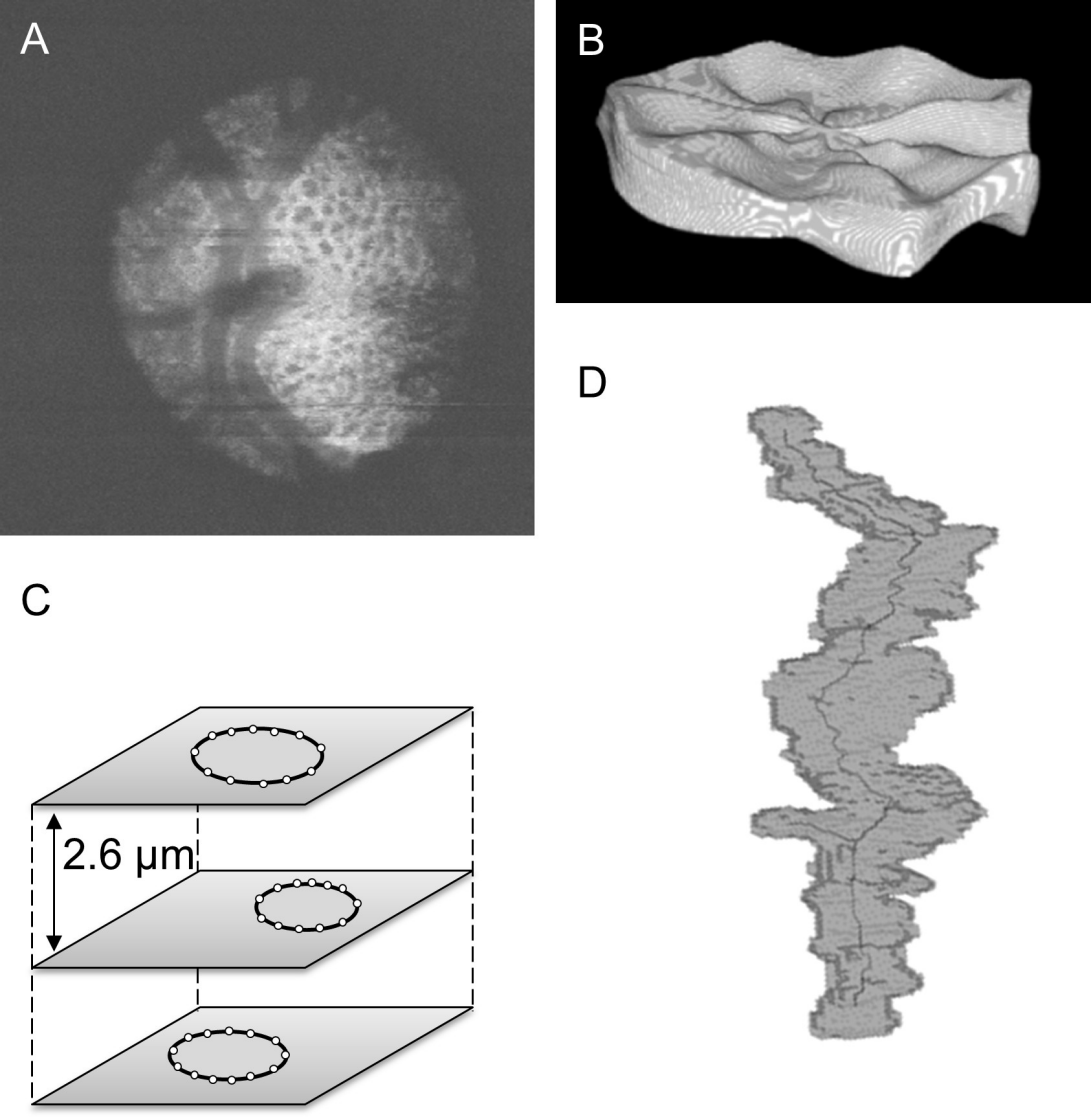
Method of segmenting the 3D structure of the laminar pores. A: En-face image of the optic nerve head based on 4x-repeated OCTA volume data. B: Automatically-segmented LC structure. C: Method for marking the outer margin of each laminar pore in sequential C-scan images at 2.6-micron intervals. D: Segmented 3D structure of the laminar pores in the same eye.

One of the goals of this study was to develop a method to quantify the 3D structure of the laminar pores. As a first step, we calculated 3D volume data from SS-OCT (Topcon) B-scans, each of which was averaged from four images acquired at the same retinal location (this method is similar to OCTA) (Fig 1A). Then, we segmented the structure of the LC in the 3D images with a previously described software technique [16]. Briefly, the entire procedure was as follows: we first scanned a 3 x 3 mm area centered on the optic disc and derived 12 reconstructed radial B-scans from these scans. We then manually marked the edges of Bruch’s membrane opening (BMO) to identify the BMO center. Next, the software synchronized the reconstructed B-scan images and a set of en-face images of the LC. The anterior and posterior borders of the LC were defined in the reconstructed B-scan images as the points where the LC pores became visible. The automatically segmented LC was preprocessed with a Gaussian filter (radius: 1) (Fig 1B) and then processed further with VCAT software (Riken, http://logistics.riken.jp/vcat/vcat/en). Using this 3D analysis software, we segmented each laminar pore in sequential C-scan images at 2.6-micron intervals. In each C-scan image, the one investigator (SM) marked several outer margin points of the laminar pores (Fig 1C) and the segmentation was confirmed by two glaucoma specialists (TN, KO) at the same time, thereby interactively segmenting the 3D structure of the laminar pores [24, 25].

### Quantification of laminar pores

After segmenting the 3D structure of the laminar pores (Fig 1D), we quantified the following six parameters of the laminar pores: 1) average cross-sectional area, calculated in all C-scan planes marked by the investigator; 2) volume, adjusted to the average thickness of the LC; 3) degree of true sphericity (ratio of the surface area of a sphere of the same volume as the laminar pores to the actual surface area of the laminar pores) [26]; 4) first principal value (P1) of the 3D structure data; 5) second principal value (P2) of the 3D structure data; and 6) third principal value (P3) of the 3D structure data. These laminar pore parameters were compared in the OAG patients and control subjects.

### Topographic map of data from C-scan images of the LC

We calculated histograms and divided the average of the cross-sectional area into 4 groups based on quartiles: severe (red), moderate (yellow), mild (green), and normal (white). Using the topographical data, we labelled each pore by superimposing the corresponding C-scan image of the LC.

### Analysis

Comparisons of pairs of groups were made with the Mann-Whitney U test, performed with SciPy on python (version 0.16.1, https://www.scipy.org/scipylib/). The Chi-square test, performed with JMP software (version 10.0.2, SAS Institute Japan Inc., Tokyo, Japan), was used for frequency data on the sex ratio. Differences between groups were assessed with the Mann-Whitney U test and differences were considered significant at *p* < 0.05.

## Results

We segmented the 3D structure of laminar pores, using a process that took more than 2 hours per one laminar pore. One laminar pore extended across approximately 90 C-scan images, with each scan image having an interval of 2.6 μm. We marked 10 points on the outer margin of the laminar pores in each C-scan image, resulting in over 900 marked points for each laminar pore. We selected 20 laminar pores in each eye, and therefore segmented a total of 160 laminar pores in the 4 healthy and 4 glaucoma eyes, requiring approximately 320 hours.

**Fig 2:**
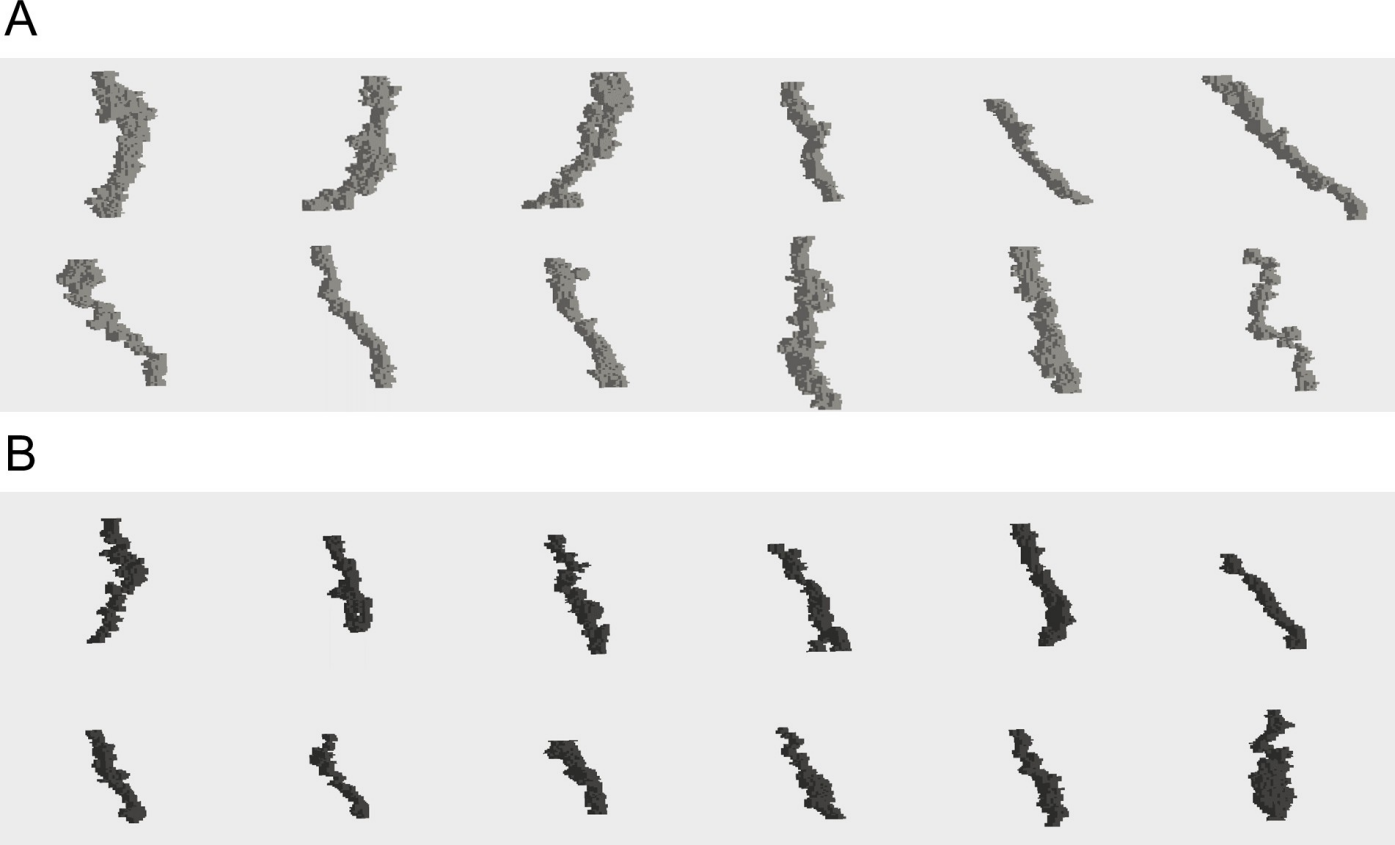
Representative 3D structure of the laminar pores.

We confirmed the segmentation accuracy of the 3D structure. Fig 2 shows a representative panel of the 3D structure of the laminar pores in 10 normal and 10 glaucoma eyes (Fig 2). The appearance of the laminar pores was smaller in the glaucoma patients.

**Fig 3:**
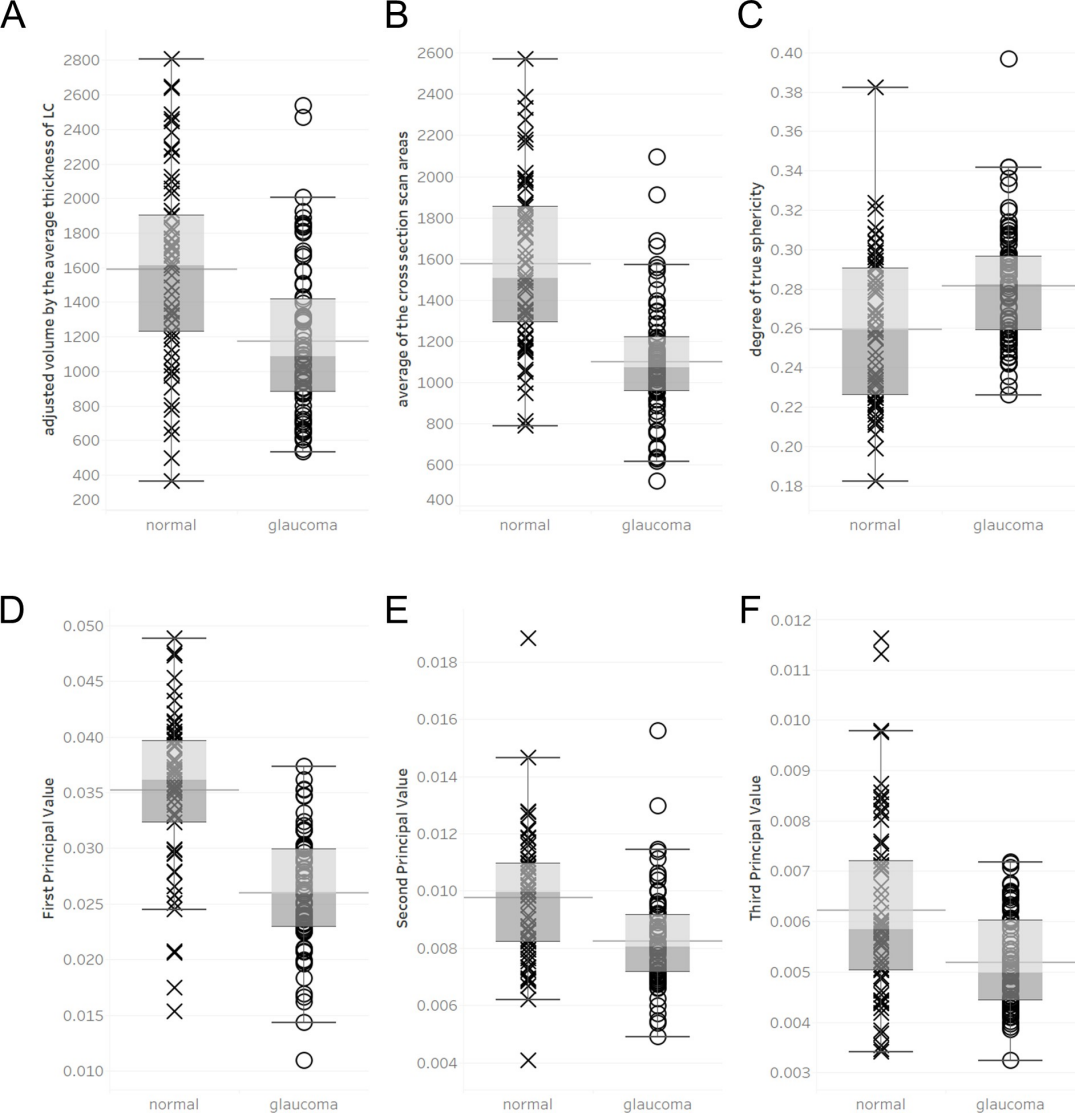
Comparison of 3D parameters of the laminar pores. Bar chart graph showing a comparison of 3D parameters. A: Average cross-sectional area of the laminar pores. B: Volume (adjusted to the lamina thickness). C: Degree of true sphericity. D: First principal value. E: Second principal value. F: Third principal value.

Statistical analysis showed that laminar pores in the OAG patients had significantly lower average cross-sectional area (normal: 1574.3 ± 401.7, glaucoma: 1101.6 ± 284.0, *p* < 0.0001), 3D volume (adjusted to the average thickness of the LC) (normal: 1590.7 ± 554.0, glaucoma: 1174.9 ± 436.4, *p* < 0.0001), first principal value (P1) (normal: 0.0352 ± 0.0071, glaucoma: 0.0260 ± 0.0052, *p* < 0.0001), second principal value (P2) (normal: 0.0098 ± 0.0023, glaucoma: 0.0083 ± 0.0017, *p* < 0.0001), and third principal value (P3) (normal: 0.0062 ± 0.0018, glaucoma: 0.0052 ± 0.0009, *p* = 0.0006). Laminar pores in the OAG patients also had a higher degree of true sphericity (normal: 0.260 ± 0.038, glaucoma: 0.282 ± 0.029, *p* = 0.0003, Fig 3).

**Fig 4:**
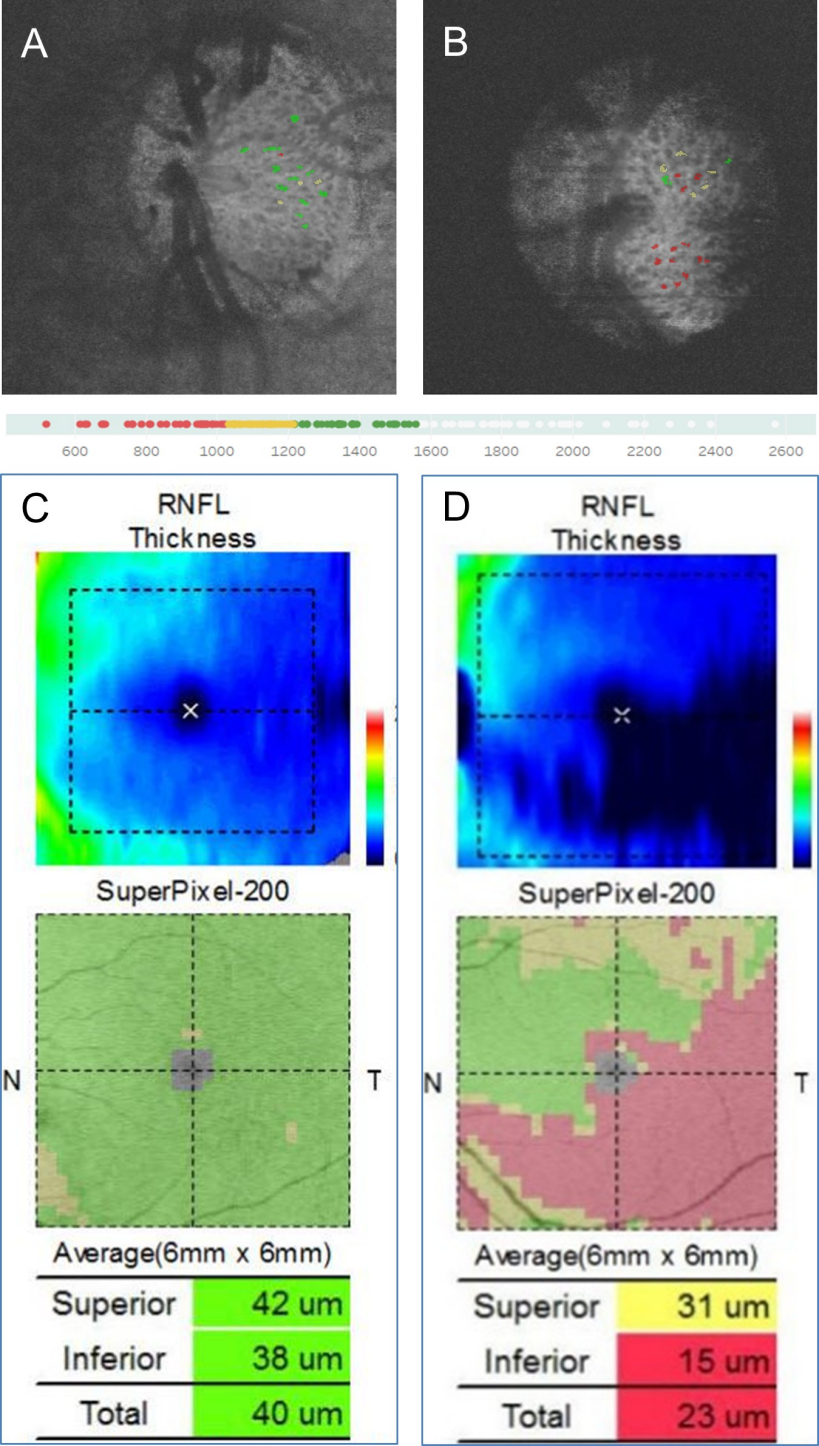
Representative cases showing the topographic distribution of damaged laminar pores. A, B: Superimposition of deviation data and en-face images of the optic nerve head. C, D: OCT macular RNFL thickness maps and OCT macular deviation maps. Representative healthy (A, C) and glaucoma (B, D) subjects. Histogram and division of the laminar pores into 4 quartile groups based on cross-sectional area: severe (red), moderate (yellow), mild (green), and normal (white).

We calculated histograms and divided the laminar pores into groups based on their average cross-sectional areas in each C-scan plane marked by the investigator. The pores were divided into 4 quartile groups: severe (red), moderate (yellow), mild (green), and normal (white). Each labeled laminar pore in the topographical data was manually superimposed on the C-scan images of the LC. Fig 4 shows representative healthy and glaucoma subjects. The damaged area of the macular map and optic nerve head was closely consistent with the topographic distribution of damaged laminar pores (Fig 4).

## Discussion

Previously, we reported that the LC becomes significantly thinner in patients with glaucoma [16]; here, we extended this finding with a description of structural changes in the laminar pores. This is a plot study with limited number of patients that was used to describe a new technique to assess substructures of the optic nerve. First, we developed a method to quantify the 3D structure of the laminar pores. Then, we compared this structure in OAG patients and normal subjects. We found that several 3D parameters of laminar pore structure decreased significantly in glaucoma, including the average cross-sectional area of the laminar pores (adjusted to the volume of lamina thickness), their degree of true sphericity, the first principal value, the second principal value, and the third principal value. Furthermore, we found that topographic data with color highlights showing the location of deviation in C-scan images of the LC were helpful for understanding the location of the damaged area in the ONH. These findings suggest that the volume of the laminar pores within the LC is significantly lower and that the true sphericity of the pores becomes deformed in glaucoma. These parameters may therefore be excellent structural biomarkers for glaucoma diagnosis. Evaluation of the laminar pores should also be helpful in investigating the pathophysiology of axonal damage in the process of glaucoma progression.

In this study, we found that the adjusted volume of the laminar pores was significantly smaller in glaucoma patients than in normal subjects. This is consistent with previous findings that with worse MD, SS-OCT-measured beam thickness to pore diameter ratio, pore diameter standard deviation, and beam thickness significantly increased, while pore diameter significantly decreased. [20] The Blue Mountains Eye Study found that the pores of the LC, as judged by stereo optic disc photographs, were commonly visible in glaucoma subjects (70.8%), but less so in normal eyes (29.3%). [18] Furthermore, a smaller mean pore area to disc area ratio is associated with a larger vertical cup to disc ratio. [27] These findings suggest that the area of the laminar pores decreases in advanced glaucoma. On the other hand, AO-SLO findings suggest that pore area is significantly larger in glaucoma than normal subjects. In a previous study, multiple regression analysis indicated that pore area was significantly correlated to axial length and untreated IOP. [19] Thus, studies using different examination methods have not consistently found that the area of the laminar pores decreases in glaucoma. However, the AO-SLO study included many patients with myopia, and the study population overall had significantly elongated axial length, which could have caused the laminar pores to have a larger area. By contrast, the present study excluded subjects with high myopia, and the influence of axial length on pore size was therefore negligible. Thus, the morphology of the laminar pores may be influenced by independent risk factors, such as elongated axial length and cupping formation.

This study also found that the area of the 3D structure of the laminar pores was smaller in the glaucoma patients. It is unclear whether this was due to axonal constriction or was simply the result of tissue remodeling following shrinking of the axonal bundles. Histopathological studies of glaucomatous damage in the ONH have revealed persistent glial activation [28] and migration [29] during chronic glaucomatous neurodegeneration, accompanied by the upregulation of the synthesis of extracellular matrix components, [28] which fill the space within the pores. To resolve these questions, it would be helpful to have more information on time-course differences during the early stages of structural changes in the laminar pores and axonal damage. Further longitudinal research is therefore needed.

One of the most important parts of this study was its demonstration of a method for 3D evaluation of laminar pore structure. All previously published reports have used 2D evaluation of laminar pore area with AO-SLO, [19] auto-segmentation of the laminar pores with SS-OCT [20, 21], or have used new types of OCT. [22] However, when the disc angle is tilted, accurate evaluation of pore structure in 2D is very difficult. We found that true sphericity and the principal value (i.e., P1, 2, and 3) of the 3D structural data were significantly different in the control and glaucoma subjects in this study. Furthermore, these parameters enabled us to assess the 3D structure of the laminar pores free of influence from tilting of the optic nerve head. This suggests that 3D evaluation will improve the accuracy of quantifications of laminar pore structure and should help clarify glaucoma pathogenesis in the near future.

The deviation map of the 3D laminar pores in the C-scan images was correlated to the location of damage in the macular thickness maps. Previously, multiple regression analysis showed that LC thickness was independently correlated to the formation of cupping and to tissue blood flow. [17] Recently, we also showed that the level of systemic oxidative stress was correlated to tissue blood flow in early glaucoma [30], while OCTA has shown that vessel index is associated with visual field loss. [31] In patients with preperimetric glaucoma (PPG), we found that tissue blood flow had already decreased, [32] and that reduced basal tissue blood flow in the ONH was associated with visual field progression. [33] Taken together, these results show that reductions in blood flow to the ONH tissue (which is fed by a branch of the short posterior ciliary artery and the circle of Zinn-Haller), might be related to changes in the laminar pores. We originally used OCTA-derived data for segmenting the 3D pore structure. Interestingly, this suggests that SS-OCT may be useful in the assessment of glaucoma-related changes in the ONH, including cupping formation, the disappearance of the capillaries in the ONH, thinning of the LC, and changes in the 3D structure of the laminar pores.

This study had several limitations. First, the number of cases included here was small. However, there were clear differences in the 3D structure of the laminar pores in the normal and glaucoma patients. There are various types of glaucomatous optic discs, and glaucoma is moreover a multifactorial disease, suggesting that further research might reveal that glaucoma patients with different disease pathogeneses might have different changes in the laminar pores. A second limitation of this study was that the analysis process relied on manually marking the images. However, to lessen any bias resulting from this, a single expert performed the marking in a blind fashion. Third, the analysis process was very time-consuming, especially the manual marking of the surface and bottom of the LC and the segmentation of the 3D laminar pore structure, which required marking up more than 90 OCT C-scan images for each pore. This process required more than a week to finish the analysis of a single case. Previously, the automated segmentation of the LC demonstrated high reproducibility for 3D LC parameters. [21] The method proposed here relies on revealing the 3D structure of each laminar pore and the development of quantitative methods. Thus, we need to develop automated or more simplified methods to investigate the relationship between 3D parameters of laminar pore structure and axonal damage in the near future. In its current form, this approach is very time consuming to be used even for research purposes in a large cohort of patients. However, the study adds to the literature and is a good starting point for future studies that will address the current limitations.

In conclusion, obtaining 4 sets of repeated OCTA scan data enabled us to calculate one set of clear volume data for the optic disc and perform segmentation of each laminar pore in glaucoma patients. We found that the 3D structure of the laminar pores in these patients was significantly smaller than normal. Thus, LC thinning, which plays a key role in glaucomatous axonal degeneration, may induce structural changes in the laminar pores. Our approach to quantitative evaluation of laminar pore structure should open new avenues for the clarification of glaucoma pathogenesis and create new fields of research into the relationship between structure and function in glaucoma.

## Supporting information

S1 File Quantitative data for six parameter of laminar pore. S1 File contains the calculated data of average cross-sectional area, volume, degree of true sphericity, first principal value (P1) of the 3D structure data, second principal value (P2) of the 3D structure data, andthird principal value (P3) of the 3D structure data.
(XLSX)
